# miR-557 inhibits hepatocellular carcinoma progression through Wnt/β-catenin signaling pathway by targeting RAB10

**DOI:** 10.18632/aging.205554

**Published:** 2024-02-15

**Authors:** Xiaoye Cheng, Can Wu, Haocheng Xu, Ruixiang Zou, Taiyuan Li, Shanping Ye

**Affiliations:** 1 Department of General Surgery, The First Affiliated Hospital, Jiangxi Medical College, Nanchang University, Nanchang 330006, Jiangxi Province, China; 2 Department of Hematology, The First Affiliated Hospital, Jiangxi Medical College, Nanchang University, Nanchang 330006, Jiangxi Province, China

**Keywords:** miR-557, RAB10, hepatocellular carcinoma, epithelial-to-mesenchymal transition, Wnt/β-catenin pathway

## Abstract

Accumulating evidence suggests that aberrant miRNAs participate in carcinogenesis and progression of hepatocellular carcinoma (HCC). Abnormal miR-557 expression is reported to interfere with the progression of several human cancers. However, the potential roles of miR-557 in HCC remain largely unknown. In the current study, we found that miR-557 was down-regulated in HCC tissues and cell lines, and was closely related to recurrence and metastasis of HCC. Notably, overexpression of miR-557 inhibited proliferation, migration, invasion, epithelial-to-mesenchymal transition (EMT) progression, blocked cells in G0/G1 phase of MHCC-97H cells *in vitro*, and suppressed tumor growth *in vivo*. However, loss of miR-557 facilitated these parameters in Huh7 cells both *in vitro* and *in vivo*. Moreover, RAB10 was identified as a direct downstream target of miR-557 through its 3’-UTR. Furthermore, RAB10 re-expression or knockdown partially abolished the effects of miR-557 on proliferation, migration, invasion, and EMT progression of HCC cells. Mechanistically, overexpression of miR-557 suppressed Wnt/β-catenin signaling by inhibiting GSK-3β phosphorylation, increasing β-catenin phosphorylation, and decreasing β-catenin transport to the nucleus, while knockdown of miR-557 activated Wnt/β-catenin signaling. Moreover, the TOP/FOP-Flash reporter assays showed that miR-557 overexpression or knockdown significantly suppressed or activated Wnt signaling activity, respectively. Additionally, low expression of miR-557 and high expression of RAB10 in HCC tissues was closely associated with tumor size, degree of differentiation, TNM stage and poor prognosis in HCC patients. Taken together, these results demonstrate that miR-557 blocks the progression of HCC via the Wnt/β-catenin pathway by targeting RAB10.

## INTRODUCTION

Most liver cancer is hepatocellular carcinoma (HCC), and HCC is an aggressive malignancy in the world [1, 2]. Although great achievements have been attained in the therapy of HCC in recent years, its prognosis is still dissatisfactory because of the high probability of recurrence and metastasis [3]. Epithelial mesenchymal transition (EMT) is a crucial procedure in the progression and metastasis of malignancies, whereby cancer cells acquire mesenchymal phenotypes and the ability to migrate and invade [4]. The occurrence of HCC is closely association with factors such as HBV/HCV infection, which involves dysregulation of many gene expression and signaling pathways [5].

microRNAs (miRNAs) are a group of mini-length and non-coding RNAs that control gene expression after transcription by binding specific mRNA, and then induce translation inhibition and/or degradation [6]. Accumulating evidence indicates that aberrant miRNAs participate in carcinogenesis and progression of HCC [7, 8]. MiR-557 was recently discovered, based on microarray data derived from gastric cancer tissues [9]. It was found to be down-regulated and acts as a tumor inhibitor in some cancer types, including pancreatic cancer [10], pancreatic ductal adenocarcinoma [11], triple-negative breast cancer [12], and lung cancer [13]. Moreover, Li et al. pointed out that its expression was decreased in HCC when studying the function of circ_0040705 [14]. In addition, Qiao et al. indicated that miR-557 could restrain the growth of osteosarcoma by regulating the expression of KRAS [15]. Another research showed that miR-557 could restrained the progression of osteosarcoma cells by targeting HOXB9 [16]. However, the potential biologic functions and possible regulatory mechanisms of miR-557 in HCC remain heavily unknown.

RAB10 belongs to the RAS oncogene superfamily with GTP and GDP binding domains [17, 18]. The RAB10 protein exists in both endocytic and exocytic compartments, and controls intracellular vesicle transporting [19]. RAB10 is a resilience locus and potential therapeutic target for Alzheimer’s disease [20]. Another study showed that RAB10 is a traffic controller in many cellular locations and pathways [21]. RAB10 can regulate intracellular logistics during neural development [22]. RAB10 participates in mediating cilia generation and cilia transport [23]. A few studies have indicated that RAB10 is overexpressed in HCC, but the biologic roles of RAB10 in HCC are still heavily unknown [24, 25].

In our study, we displayed that miR-557 was low expressed in both cells and tissues of HCC, and was compactly related to HCC recurrence and metastasis. MiR-557 acted as a suppressor of HCC progression. Moreover, we found that miR-557 inhibited Wnt/β-catenin pathway by stimulating β-catenin phosphorylation and decreasing β-catenin shift to the nucleus, which was mediated by inhibited RAB10. Additionally, down expression of miR-557 and high expression of RAB10 in HCC was bound up with malignant clinical characteristics and poor prognosis. The results of our study offer a new insight into the regulatory mechanisms of miR-557 in HCC progression.

## RESULTS

### MiR-557 down-regulation in HCC tissues predicts poor prognosis and metastasis of HCC

**Figure 1:**
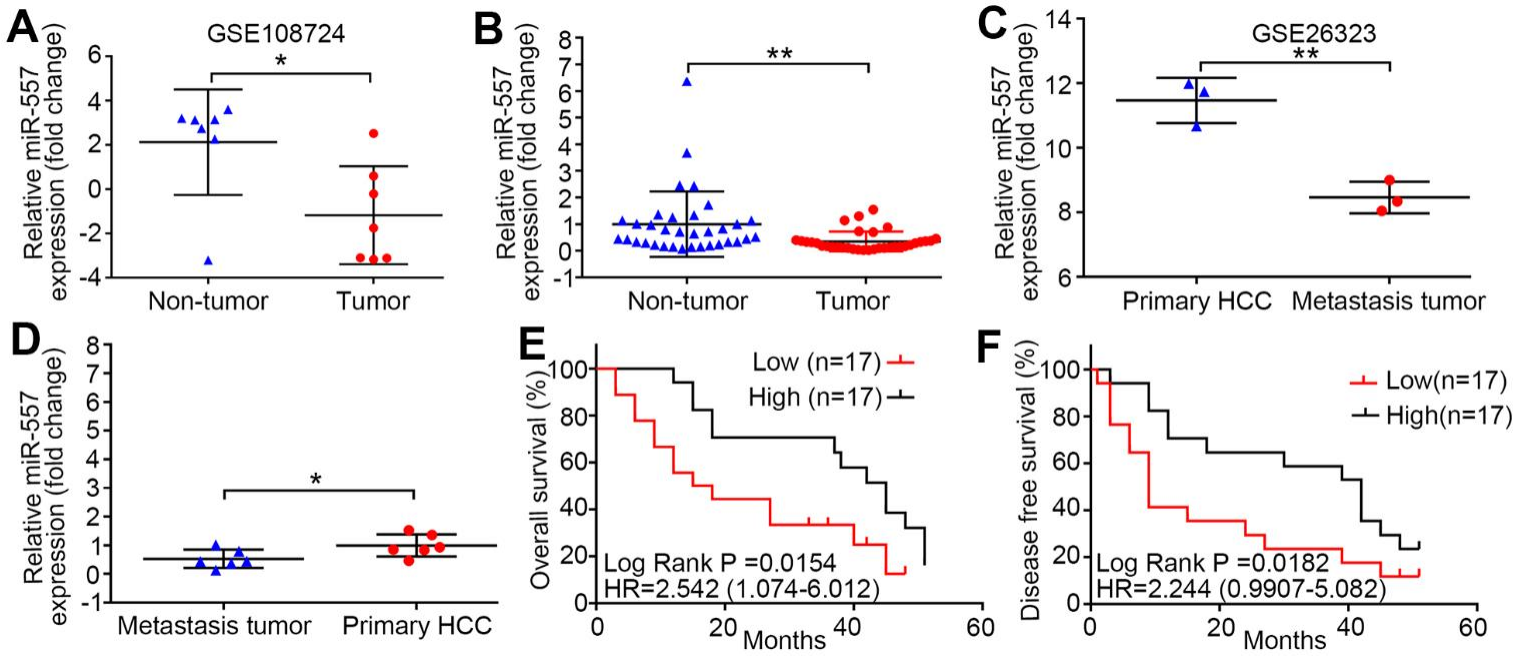
**MiR-557 down-regulation in HCC predicts poor prognosis and metastasis of HCC.** (**A**) Relative expression of miR-557 in HCC tissues and adjacent non-tumor tissues of GEO database (GSE108724). (**B**) Relative miR-557 expression in HCC tissues and adjacent non-tumor tissues. (**C**) Relative expression of miR-557 in primary HCC tissues and pulmonary metastasis of HCC tissues of GEO database (GSE26323). (**D**) Relative miR-557 expression in primary HCC tissues and paired pulmonary metastasis of HCC tissues. (**E**) The relationship between the expression level of miR-557 and overall survival of HCC patients. (**F**) The relationship between the expression level of miR-557 and disease-free survival of HCC patients. (^*^P<0.05, ^**^P<0.01).

Using the GEO database (GSE108724), we first compared the miR-557 expression level between HCC and contiguous non-cancer tissues. The level of miR-557 expression was lower in HCC than in contiguous non-cancer (Figure 1A). We detected the miR-557 expression level in 34 paired HCC and contiguous non-cancer tissues in our center and discovered that the mean expression was also down-regulated in HCC than that in contiguous non-cancer tissues (Figure 1B). To deeply investigate the potential function of miR-557 in HCC metastasis, we searched the GEO database for HCC metastasis (GSE26323) and compared the miR-557 expression level in pulmonary metastasis of HCC tissues and HCC tissues. The mean expression of miR-557 was lower in pulmonary metastasis tissues than in primary HCC tissues (Figure 1C). We also checked the miR-557 expression level in 6 paired pulmonary metastasis of primary HCC tissues and HCC tissues in our center. We showed that miR-557 was down-expression in pulmonary metastasis of primary HCC tissues than in HCC tissues in 6 patient specimens (Figure 1D). Moreover, we look into the relation between miR-557 expression and survival of patients. The results indicated that patients with lower miR-557 expression level showed significantly worse overall survival (OS) than patients with high miR-557 level (*P* = 0.0154) (Figure 1E). The HCC patients with lower miR-557 expression level exhibited shorter disease-free survival (DFS) than HCC patients with up-regulated miR-557 expression level (*P* = 0.0187) (Figure 1F).

We far explored the relation between miR-557 expression level and clinicopathological factors. The results indicated that patients with down-regulated miR-557 in HCC tissues were significantly associated with larger tumors than patients with miR-557 overexpression (*P* = 0.001) (Table 1). Compared to HCC suffers from an up expression of miR-557, HCC suffers with a lower expression of miR-557 showed poor tumor differentiation (*P* = 0.039) (Table 1). For tumor staging, HCC patients with lower expression of miR-557 exhibited relatively advanced AJCC TNM staging (*P* = 0.001) (Table 1). All in all, these results demonstrate that miR-557 may play a vital biological role in HCC.

**Table 1:** Clinicopathological features of HCC patients between miR-557 high- and low-expression cohorts.

**Features**	**qRT-PCR**		***P*-value**
	**Low (n = 17)**	**High (n = 17)**	
Gender, n			0.656
Male	13	15	
Female	4	2	
Age, years			1.000
≤ 50	8	8	
> 50	9	9	
HBV infection, n			0.227
Positive	14	17	
Negative	3	0	
AFP, ng/ml			1.000
≤ 400	10	9	
> 400	7	8	
Tumor number, n			1.000
Solitary	16	15	
Multiple	1	2	
Tumor size, cm			0.001*
≤ 5	5	15	
> 5	12	2	
Vascular invasion, n			0.491
Yes	6	9	
None	11	8	
Tumor differentiation, n			0.039*
Well/moderate	10	16	
Poor	7	1	
Liver cirrhosis, n			0.708
Yes	13	11	
None	4	6	
AJCC TNM stage, n			0.001*
I/II	5	15	
III/IV	12	2	

AFP, Alpha-fetoprotein; AJCC*,* American Joint Committee on Cancer; HBV, Hepatitis B virus; HCC, Hepatocellular Carcinoma; *P<0.05. Fisher’s exact test was used in all analyses.

### MiR-557 inhibits HCC cell growth, suppresses invasion and migration, induces cell cycle arrest, and reverses EMT

**Figure 2:**
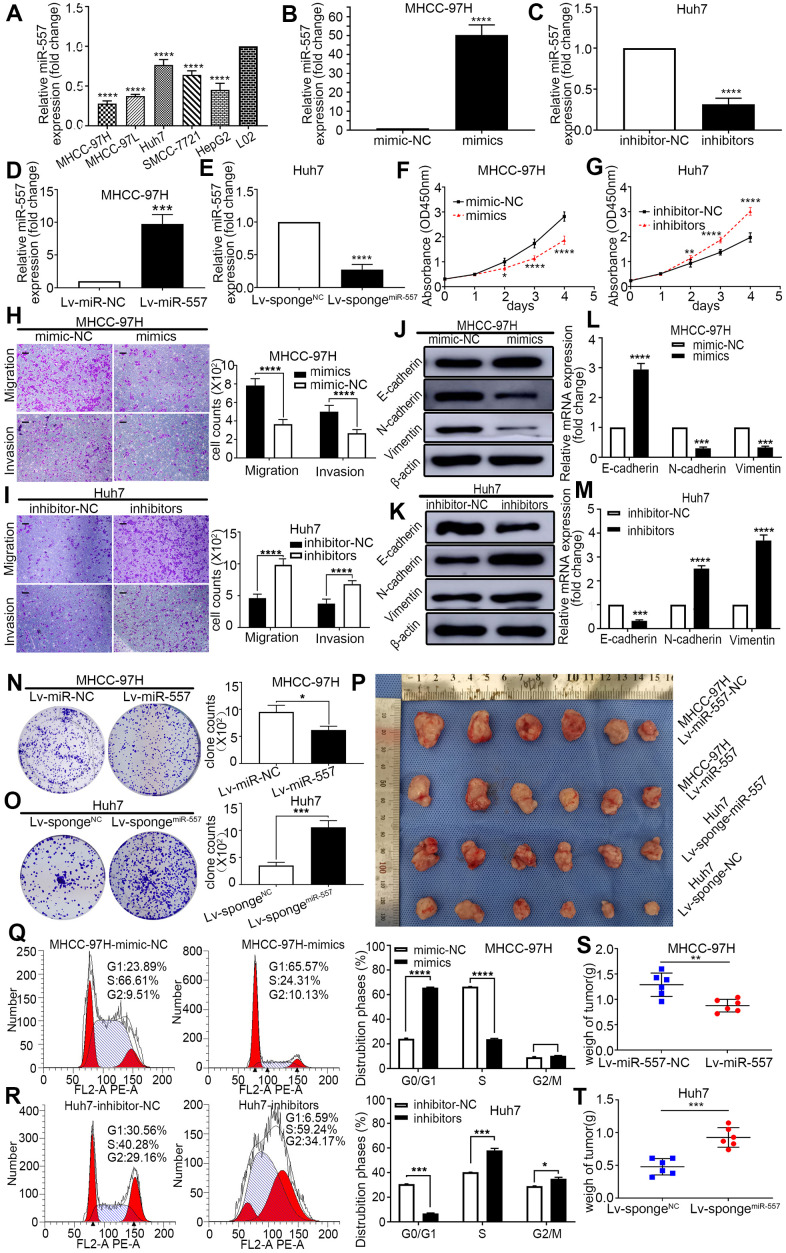
**Effect of miR-557 on HCC cell proliferation, migration, invasion, cell cycle, and EMT.** (**A**) Relative expression of miR-557 in different HCC cells lines. (**B**–**E**) Relative miR-557 expression in HCC cells that overexpressed miR-557, in MHCC-97H cells, and knockdown miR-557 in Huh7 cells. (**F**, **G**) CCK8 assays of mimic-NC cells and miR-557-overexpression cells of MHCC-97H, and CCK8 assays of inhibitor-NC cells and miR-557-inhibition cells of Huh7 cells. (**H**, **I**) Transwell assays of mimic-NC cells and miR-557-overexpression cells of MHCC-97H, and transwell assays of inhibitor-NC cells and miR-557-inhibition cells of Huh7 cells, Scale bar: 100 μm. (**J**–**M**) The protein and mRNA of EMT process of mimic-NC cells and miR-557-overexpression cells of MHCC-97H, and of inhibitor-NC cells and miR-557-inhibition cells of Huh7 cells. (**N**, **O**) Clone formation assay of Lv-miR-NC cells and Lv-miR-557-overexpression cells of MHCC-97H, and Clone formation assay of Lv-sponge-NC cells and Lv-sponge-miR-557-inhibition cells of Huh7 cells. (**P**) Orthotopic transplantation tumor formation with Lv-miR-NC and Lv-miR-557 overexpression in MHCC-97H cells or Lv-sponge-NC and Lv-sponge-miR-557-inhibition cells of Huh7 cells. (**Q**, **R**) The distribution phases of cell cycle of mimic-NC cells and miR-557-overexpression cells of MHCC-97H, and of inhibitor-NC cells and miR-557-inhibition cells of Huh7 cells. All blots were cut prior to hybridisation with antibodies during western blotting. (**S**, **T**) The tumor weight analysis of mimic-NC cells and miR-557-overexpression cells of MHCC-97H, and of inhibitor-NC cells and miR-557-inhibition cells of Huh7 cells. (*P < 0.05, **P < 0.01, ***P < 0.001, ****P < 0.0001).

To go deeply into the biological function of miR-557 on HCC, we examined the miR-557 expression in HCC cell lines and normal liver cell line L02. MiR-557 expression was relatively lower in HCC cell lines than in normal liver cell line L02 (Figure 2A). We over expressed miR-557 expression by transducing mimics or lentiviruses into MHCC-97H cells and down-regulated miR-557 expression using inhibitors or transducing lentiviruses into Huh7 cells (Figure 2B–2E). CCK-8 assays displayed that overexpression of miR-557 inhibited HCC proliferation, whereas down-regulation of miR-557 promoted HCC proliferation in comparison with the control cells (Figure 2F, 2G).

Based on the transwell experiments, miR-557 overexpression significantly restrained the migration and invasion skills of HCC cells, and knockdown of miR-557 enhanced the migration and invasion skills of HCC cells (Figure 2H, 2I). Next, we look into the impact of miR-557 on the EMT of HCC since EMT is a vital step in tumor migration and invasion [4]. We detected the relationship of miR-557 and biomarkers of EMT in transfected HCC cells. The outcomes manifested that the expression level of miR-557 was oppositely associated with the N-cadherin and Vimentin (mesenchymal markers) but positively correlated with the E-cadherin (epithelial markers) (Figure 2J–2M).

To go a step further investigate the impact of miR-557 on the proliferation of HCC, we conducted colony formation assays. The outcomes revealed that up-expression of miR-557 inhibited colony formation ability whereas down-regulation of miR-557 promoted colony formation ability in comparison with control cells (Figure 2N, 2O). Moreover, to deeper look into the role of miR-557 in HCC progression, we utilized the orthotropic transplantation model in BALB/c nude mice. We assessed the tumor sizes of these mice with HCC transfected cells. Tumor sizes in the miR-557 overexpression group were obviously light weight than that in the control group (Figure 2P–2S). The miR-557 knockdown group had significantly larger sizes of orthotropic transplantation tumors (Figure 2P, 2T).

Both cell proliferation and differentiation are regulated by the cell cycle [26]. As shown in Figure 2Q, 2R, up-expression of miR-557 could block HCC cells in the G0/G1 period while knockdown of miR-557 could change the cell cycle stage from G0/G1 till G2/M. Collectively, the aforementioned results imply that miR-557 acts a momentous impact in the progression of HCC.

### MiR-557 binds to the 3’-UTR of RAB10

**Figure 3:**
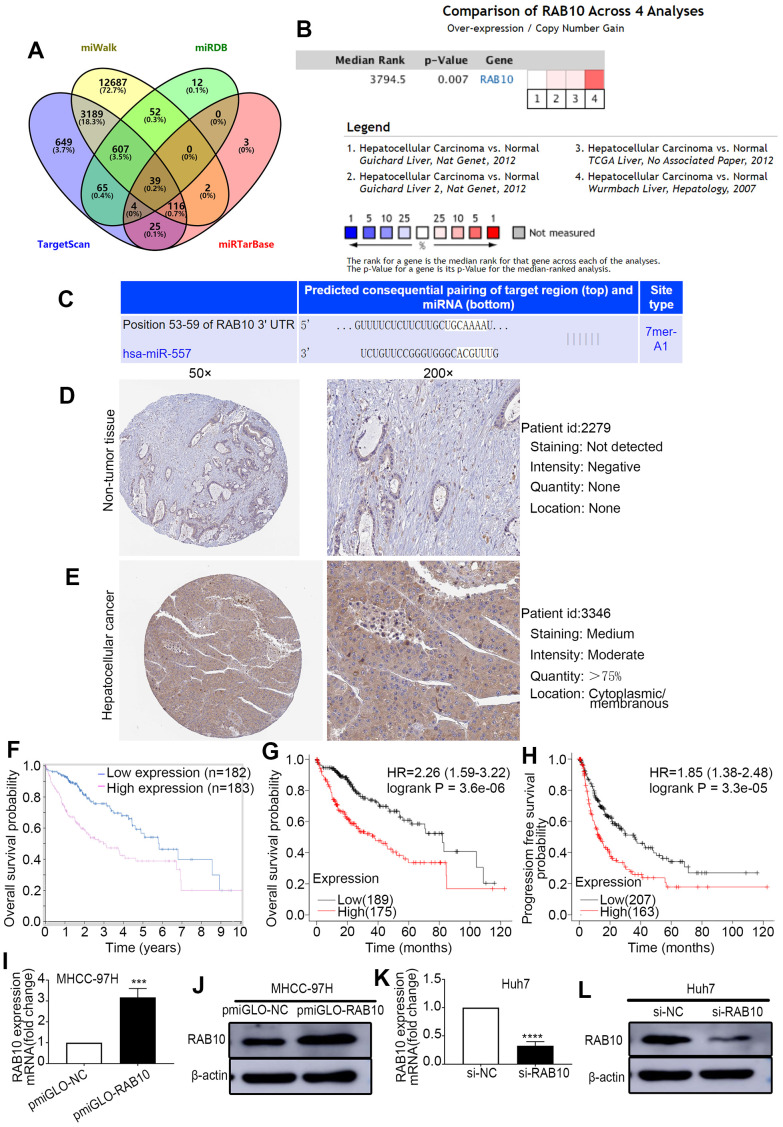
**RAB10 is a downstream target gene of miR-557, is overexpressed in HCC, and is related to poor prognosis.** (**A**) Venn diagram of TargetScan, miWalk, miRDB, and miRTarBase. (**B**) Meta analysis of RAB10 expression from Oncomine database. (**C**) Predicted binding sites of 3′-UTR of RAB10 to miR-557. (**D**, **E**) The immunohistochemical images of relative RAB10 expression in HCC tissues and paired non-tumor tissues stem from the Human Protein Atlas database (Version 23.0, https://www.proteinatlas.org/ENSG00000084733-RAB10/pathology/liver+cancer#img). (**F**) The relationship between RAB10 expression and overall survival from the Human Protein Atlas database. (**G**, **H**) The relationship between the expression level of RAB10 and overall survival or disease-free survival from the Kaplan–Meier plotter online database. (**I**–**L**) The transfection efficiency of pmirGLO-NC, pmirGLO-RAB10 of MHCC-97H cells, and of siNC and siRAB10 of Huh7 cells. All blots were cut prior to hybridisation with antibodies during western blotting. (^***^P<0.001, ^****^P<0.0001).

To explore the specific target gene of miR-557 through which it might lead to tumor suppression gene behavior *in vivo* and *in vitro*, public online bioinformatics databases (TargetScan, miRDB, miWalk and miRTarBase) were used. Out of 39 potential predicted targets (Figure 3A), four potential target genes (ARID1A, WAC, CALM2, RAB10) were selected not only because they were highly expressed in both the Online and ULACAN databases, but also because they had a significant impact on survival in both the ULACAN and Kaplan–Meier plotter online database (Supplementary Table 2). RAB10 was selected not only because it was deemed as an oncogene (Figure 3B) [25] but also for its relatively high score of binding sites (Figure 3C), and it has a greater impact on survival in both the ULACAN and Kaplan–Meier plotter online database.

Firstly, we look into the expression level and the prognosis value of RAB10 in the online database. The data stem from the Oncomine database indicated that the expression of RAB10 gene was more in HCC than in non-cancer tissues (Figure 3B). Analysis of the Human Protein Atlas database (Version: 23.0) also found that RAB10 protein expression was more in HCC than in non-cancer tissues (Figure 3D, 3E), the immunohistochemical images available from the Human Protein Atlas (https://www.proteinatlas.org/ENSG00000084733-RAB10/pathology/liver+cancer#img). Moreover, prognostic analysis using both the Human Protein Atlas database and the Kaplan–Meier plotter database displayed that up-expression of RAB10 was relevant to poor prognosis of HCC (Figure 3F–3H).

**Figure 4:**
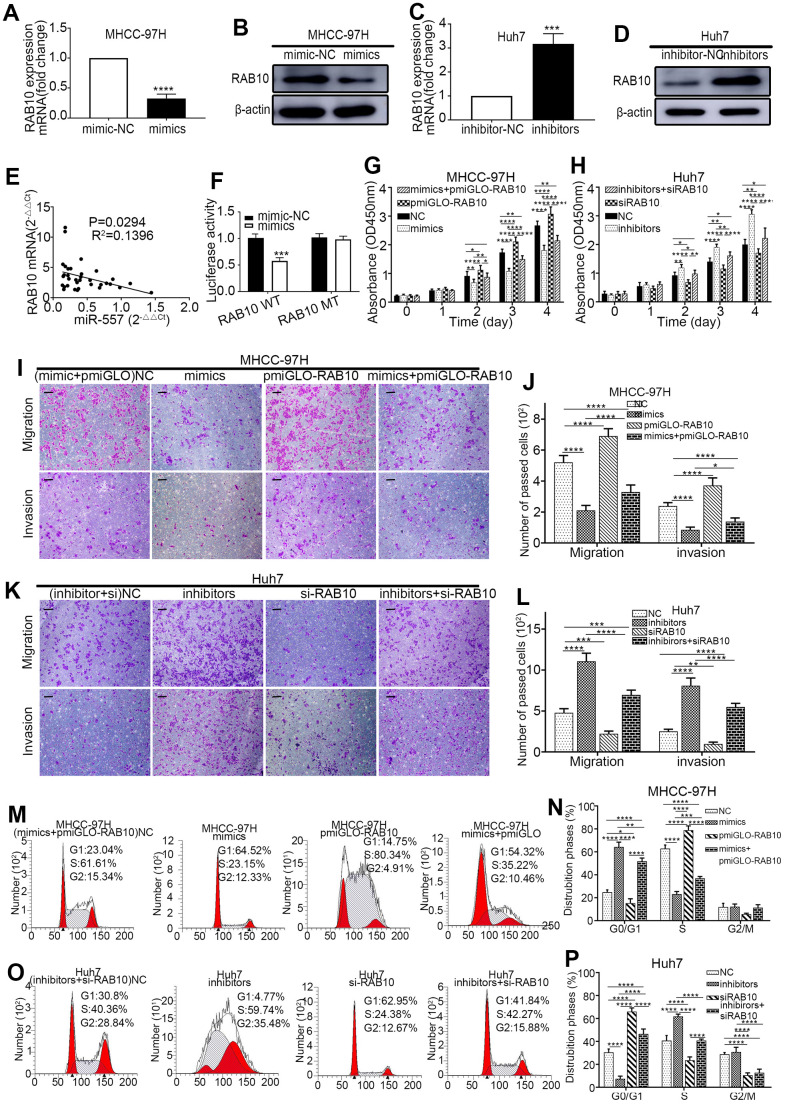
**MiR-557 targets RAB10 to affect the biological behaviour of cells.** (**A**–**D**) Relative expression of RAB10 in mimic-NC cells and miR-557-overexpression cells of MHCC-97H, and in inhibitor-NC cells and miR-557-inhibition cells of Huh7 cells. (**E**) The relationship between the expression level of miR-557 and the expression level of RAB10 in patients. (**F**) The relative dual-luciferase reporter assays in four different groups. (**G**) CCK-8 assays of NC cells, miR-557-overexpression cells, RAB10-overexpression cells, co-transfected miR-557 mimics and pmirGLO-RAB10 cells of MHCC-97H. (**H**) CCK-8 assays of NC cells, miR-557-down-expression cells, RAB10-down-expression cells, co-transfected miR-557 inhibitors and siRAB10 cells of Huh7. (**I**, **J**) Transwell assays of NC cells, miR-557-overexpression cells, RAB10-overexpression cells, co-transfected miR-557 mimics and pmirGLO-RAB10 cells of MHCC-97H, Scale bar: 100 μm. (**K**, **L**) Transwell assays of NC cells, miR-557-down-expression cells, RAB10-down-expression cells, co-transfected miR-557 inhibitors and siRAB10 cells of Huh7, Scale bar: 100 μm. (**M**, **N**) The distribution phases of cell cycle of NC cells, miR-557-overexpression cells, RAB10-overexpression cells, co-transfected miR-557 mimics and pmirGLO-RAB10 cells of MHCC-97H. (**O**, **P**) The distribution phases of cell cycle of NC cells, miR-557-down-expression cells, RAB10-down-expression cells, co-transfected miR-557 inhibitors and siRAB10 cells of Huh7. All blots were cut prior to hybridisation with antibodies during western blotting. (^*^P<0.05, ^**^P<0.01, ^***^P<0.001, ^****^P<0.0001).

To attest the relevance between miR-557 and RAB10 in HCC, Western blot and RT-qPCR assays were implemented. The outcomes exhibited that the expression of RAB10 was lower in the groups of HCC cells infected with miR-557 mimic than those infected with miR-mimic-NC (Figure 4A, 4B). Similarly, the level of RAB10 expression was higher in the groups of HCC cells infected with miR-557 inhibitor than those infected with miR-inhibitor-NC (Figure 4C, 4D). We also look into the expression levels of miR-557 and RAB10 genes in the clinical samples in our center using RT-qPCR and discovered that the expression of miR-557 adversely relevant with the expression of RAB10 (Figure 4E).

To vindicate that RAB10 is a downstream gene of miR-557, luciferase reporter test was conducted. The luciferase activity in the group of pmirGLO-RAB10-WT and miR-557 mimics co-transfected was suppressed compared with that in the group of pmirGLO-RAB10-WT and miR-557-mimic-NC co-transfected. However, the luciferase activity in the group of pmirGLO-RAB10-MT and miR-557 mimics co-transfected was similar to that in the group of pmirGLO-RAB10-MT and miR-557-mimic-NC co-transfected (Figure 4F).

### RAB10 mediated the impact of miR-557 on the migration, proliferation, colony formation, invasion, cell cycle and EMT of HCC cells

**Figure 5:**
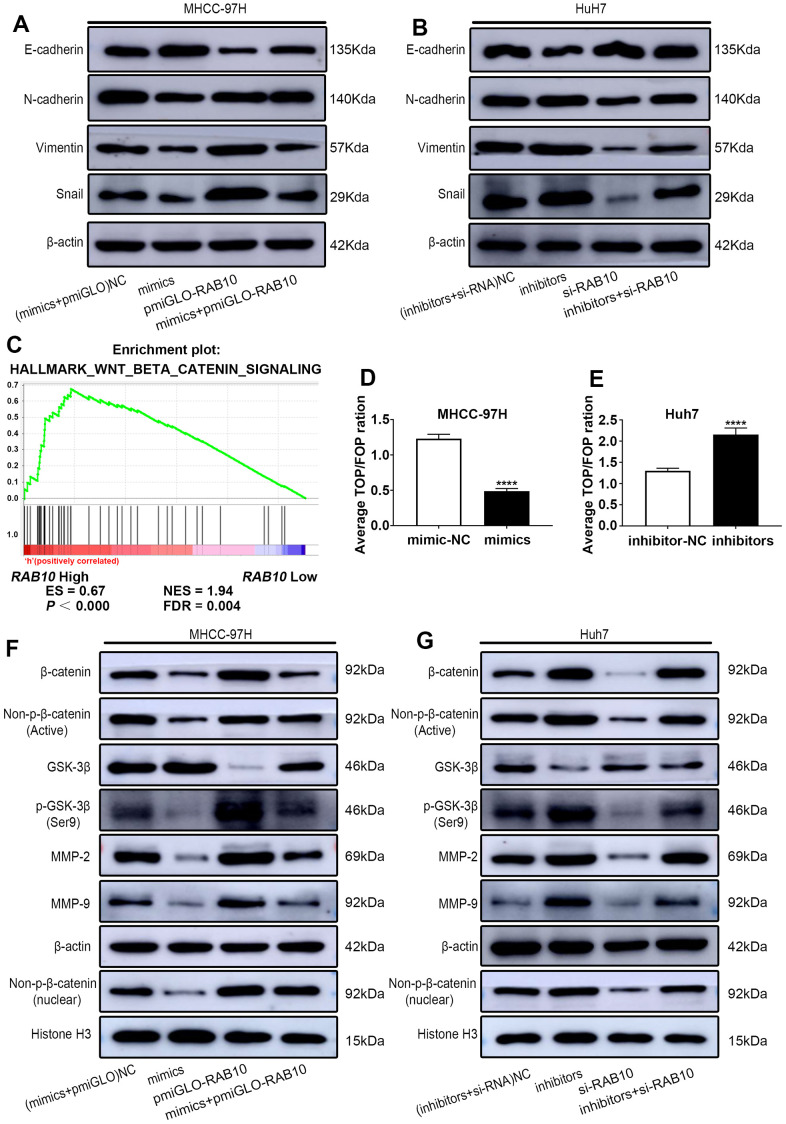
**MiR-557 targets RAB10 to affect the process of EMT and inhibit Wnt/β-catenin signaling.** (**A**) The protein of EMT process of NC cells, miR-557-overexpression cells, RAB10-overexpression cells, co-transfected miR-557 mimics and pmirGLO-RAB10 cells of MHCC-97H. (**B**) The protein of EMT process of NC cells, miR-557-down-expression cells, RAB10-down-expression cells, co-transfected miR-557 inhibitors and siRAB10 cells of Huh7. (**C**) GSEA of RAB10 expression and Wnt/β-catenin signaling. (**D**, **E**) TOP-flash/FOP-flash assays of mimic-NC cells and miR-557-overexpression cells of MHCC-97H, and TOP-flash/FOP-flash assays of inhibitor-NC cells and miR-557-inhibition cells of Huh7 cells. (**F**) Western blot analysis of AKT/FOXO3a signaling-related proteins in NC cells, miR-557-overexpression cells, RAB10-overexpression cells, co-transfected miR-557 mimics and pmirGLO-RAB10 cells of MHCC-97H. (**G**) Western blot analysis of AKT/FOXO3a signaling-related proteins in NC cells, miR-557-down-expression cells, RAB10-down-expression cells, co-transfected miR-557 inhibitors and siRAB10 cells of Huh7. All blots were cut prior to hybridisation with antibodies during western blotting. (^****^P<0.0001).

Previous studies have informed that RAB10 can promote the colony formation, and cell cycle progression, proliferation of HCC cells [25]. The luciferase reporter tests indicated that RAB10 is a downstream target gene of miR-557. We hypothesized that altering RAB10 expression could turn the effect of miR-557 on HCC. Western blot and RT-qPCR were used to test the infection efficiency of pmirGLO-RAB10 and siRAB10 (Figure 3I–3L). To verify this, we co-transfected miR-557 inhibitors with siRAB10 into Huh7 cells and co-transfected miR-557 mimics with pmirGLO-RAB10 into MHCC-97H cells. CCK-8, transwell, cell cycle, and EMT assays showed that silencing RAB10 expression partially blocked the furtherance impact of miR-557 inhibitors on invasion, proliferation, and migration of Huh7 cells whereas up-regulating RAB10 partially turn the suppression impact of miR-557 mimics on proliferation (Figure 4G, 4H), invasion and migration (Figure 4I–4L), cell cycle (Figure 4M–4P), and EMT (Figure 5A, 5B) of MHCC-97H cells. These results manifested that RAB10 acts as a target member of miR-557 and plays a crucial component in the invasion, proliferation, migration, cell cycle regulation, and EMT process of HCC cells.

### Wnt/β-catenin signaling is inhibited by miR-557

In order to further understand the regulatory mechanisms behind the miR-557/RAB10 axis on HCC, we explored the downstream signaling pathway of miR-557/RAB10 axis using GSEA. The level of RAB10 expression was correlated positively with Wnt/β-catenin pathway activity (Figure 5C), suggesting RAB10 might regulate Wnt/β-catenin pathway. The TOP/FOP luciferase activity test was selected to determine whether miR-557 can regulate Wnt/β-catenin pathway. The outcomes turned out that miR-557 overexpression inhibited Wnt/β-catenin pathway, but miR-557 knockdown significantly activated Wnt/β-catenin pathway (Figure 5D, 5E). β-catenin is a crucial component of the Wnt/β-catenin pathway and acts a pivotal role in carcinogenesis [27]. Western blot assays manifested that miR-557 up-expression significantly decreased β-catenin expression whereas RAB10 overexpression obviously increased β-catenin expression (Figure 5F). Transfecting pmirGLO-RAB10 into miR-557-overexpressed MHCC-97H cells partially abolished the impacts of miR-557 on β-catenin expression (Figure 5F). The expression level of non-p-β-catenin, the activated form of β-catenin, reflects the activity of the pathway [27, 28]. As shown in figure 5F, whether transfected with miR-557 mimics or pmiGLO-RAB10, the change trend of non-p-β-catenin was similar to β-catenin.

GSK-3β combines with other factors to form a destructive complex that plays a momentous role in promoting the degradation of β-catenin in the Wnt pathway [29, 30]. The data from our western blot assays suggested that miR-557 mimics could ascend the expression of GSK-3β protein in MHCC-97H cells, and pmirGLO-RAB10 could partially weaken the role of miR-557 on the expression of GSK-3β (Figure 5F). When GSK-3β is phosphorylated at site Ser9, β-catenin is released from the destructive complex to avoid degradation [31]. The outcomes manifested that miR-557 mimics could down-regulate the expression of p-GSK-3β (Ser9) protein, and that pmirGLO-RAB10 could partially block the functions of miR-557 on the expression of p-GSK-3β (Figure 5F). Non-p-β-catenin protein can function only when it is transferred into the nucleus [31, 32]. Hence, we detected the expression of non-p-β-catenin protein in the nucleus, and found that although miR-557 mimics could decrease its expression, re-expression of RAB10 could partially weaken the roles of miR-557 on HCC (Figure 5F).

Moreover, we further assessed the change trend of migration- and proliferation-related proteins in response to miR-557. The outcomes illustrated that miR-557 mimics inhibited the expression of MMP2/9, and re-expression of RAB10 enhanced the expression of MMP2/9 and partially weakened the roles of miR-557 on HCC (Figure 5F). However, after transfecting miR-557 inhibitors and si-RAB10, the above proteins showed the opposite trend (Figure 5G). In addition, transfecting si-RAB10 into miR-557 down-expressed Huh7 cells partially abolished the impacts of miR-557 on Wnt/β-catenin signaling (Figure 5F). All in all, the results indicate that miR-557 inhibits Wnt/β-catenin signaling.

## DISCUSSION

Although a lot of researches have demonstrated that miRNAs play a key part in the progression of HCC, it is still the tip of the iceberg for better understanding the mechanism of progression of HCC [33]. In this study, miR-557 was found to have significantly low expression in HCC tissues and cells, miR-557 expression negatively with tumor size, AJCC tumor staging, and RAB10 expression while positively correlation with degree of differentiation of HCC. Moreover, experiments have suggested that miR-557 inhibits cell proliferation, invasion, migration, EMT, and induces cell cycle arrest. RAB10 was first confirmed as a target gene of miR-557 through its 3’-UTR. We also discovered that miR-557 performs its biological roles through the Wnt/β-catenin pathway.

To date, studies on the relationship between miR-557 and tumors are limited [9–13], while the biological impacts and potential molecular mechanisms of miR-557 in HCC remain unresearched. In this exploration, we not only found that miR-557 had low expression in HCC cells and tissues, but also found the same in pulmonary metastasis HCC tumors. MiR-557 was found to be associated with malignant characteristics of tumors, and the DFS and OS of HCC patients in the group with down miR-557 expression were dramatically shorter than those in the high miR-557 expression group. Based on aforementioned experiments, it was found that up-regulation of miR-557 inhibited tumor migration, growth, proliferation, invasion, the process of EMT, and interrupted the cell cycle at the G0/G1 stage, whereas down-regulation of miR-557 showed the opposite trend. These data manifested that miR-557 may work as a cancer suppressive factor in HCC, and abnormal low expression of miR-557 may be a poor prognosis predictor in HCC patients.

miRNAs conduct their intentions by operating the expression of downstream genes. The current study identified the tumor suppressor function of miR-557 in HCC. However, the potential mechanism of action of miR-557 in HCC remained unclear. Thus, we applied the TargetScan, miRTarBase, miRDB, and miRWalk databases to calculate the potential downstream target gene of miR-557 and found RAB10 may be its candidate target gene. Then, we found a opposite correlation between miR-557 and RAB10 expression in both clinical specimens and HCC cells transfected with mimics/inhibitors. Moreover, the outcomes from the luciferase reporter assay attested that miR-557 regulated RAB10 by acting on its 3’-UTR. To further confirm whether the biological functions of miR-557 on cells were through inhibiting RAB10 expression, we up/down-regulated RAB10 expression in miR-557-overexpressing/miR-557-silencing cells and checked the biological behavior of cells. The results suggested that changing the expression of RAB10 could partially turn the impacts of miR-557 on the migration, proliferation, invasion, cell cycle regulation, and EMT procedure of HCC cells. Research has displayed that RAB10 plays a considerable role in tumor progression as a cancer oncogene [34]. RAB10 expression is increased in various malignancies such as stomach cancer, cervical cancer, osteosarcoma, glioma, and HCC. The over-expression of RAB10 is concern with poor patient prognosis and aggressive clinical features [24, 34–37]. The results of our investigation were in line with these previous studies.

The potential mechanism of the miR-557/RAB10 axis in inhibiting the invasion, proliferation, cell cycle regulation, migration, and EMT procedure of HCC remains unclear. GSEA studies indicated that the Wnt/β-catenin pathway is crucial, along with the changes in RAB10 expression. The Wnt/β-catenin pathway has been widely implicated in human cancers [32]. Anomalous activation of Wnt/β-catenin pathway is closely concern with the occurrence, malignant progression, and poor prognosis of cancer [32]. In Wnt/β-catenin pathway, β-catenin serves as an intracellular signal transducer and plays an important motive in tumorigenesis [28]. In the current exploration, up-regulation of miR-557 expression reduced the expression level of β-catenin and inhibited non-p-β-catenin transfer into nucleus whereas down-regulation of miR-557 showed the opposite trend. In addition, GSK-3β is also tightly linked with Wnt/β-catenin pathway, by affecting the stability of β-catenin [38]. In our study, up-regulation of miR-557 expression increased the expression level of GSK-3β and reducing phosphorylation of GSK-3β, whereas down-regulation of miR-557 showed the opposite effects. Moreover, silencing or up-regulating RAB10 partially abolished the impact of miR-557 on Wnt/β-catenin pathway.

Changes in the cell cycle are closely concern with cell proliferation and growth [26]. Our results suggested that up-regulation of miR-557 expression induced cell cycle discontinue at the G0/G1 while decline of miR-557 expression promoted cell cycle from G0/G1 to G2/M. As expected, cell cycle changes were consistent with cell proliferation data in our study. EMT is a momentous step in the migration, invasion and distant metastasis of cancers [39]. Snail is a classic EMT-induced transcription factor. Our study indicates that miR-557 can inhibit invasion and migration of HCC cells, by restraining the process of EMT. Lei et al. also reported that miRNAs target the Wnt/β-catenin pathway to regulate EMT in cancer [40]. Therefore, we conclude that miR-557 may regulate the biological behaviour of HCC cells via Wnt/β-catenin pathway.

In summary, for the first time, we have demonstrated that the biological impacts of miR-557 in HCC. We discovered that miR-557 inhibits proliferation, migration, invasion, EMT, and discontinues the cell cycle at the G0/G1 stage via the RAB10/Wnt/β-catenin pathway. The epigenetic regulation of microRNAs demonstrates that these molecules have great therapeutic potential in regulating the genetic activity of different cancers, including HCC, RNA based therapy does not alter the genetic code in cancer cells, but can control oncogenes and/or reactivate suppressed tumor suppressor genes, thereby achieving anti-tumor effects [41]. Thus, above results may provide new predictive therapeutic genes and indicators for HCC treatment.

## CONCLUSIONS

In this study, our data manifested that low levels of miR-557 are expressed in HCC cells and tissues, especially in pulmonary metastasis HCC tumors. MiR-557 expression negatively correlates with tumor size and AJCC tumor staging of HCC, but positively correlates with degree of differentiation and worse prognosis in patients. Based on aforementioned assays, we confirmed that miR-557 inhibits proliferation, migration, invasion, EMT, and discontinues the cell cycle at the G0/G1 stage via the RAB10/Wnt/β-catenin pathway. Therefore, our results deemed that miR-557 may be a new predictive factor of prognosis in patients suffer with HCC and may be a prospectively target for the therapy of HCC.

## MATERIALS AND METHODS

### Clinical specimens

HCC and matched contiguous non-cancer tissues were retrieved from 34 HCC patients between October 2017 and January 2018 in our center. Another six HCC specimens, including pulmonary metastasis of primary HCC tissues, HCC tissues, and matched contiguous non-cancer tissues, were gathered between June 2018 and December 2020 in our hospital. All fresh HCC and contiguous non-tumor tissues were stored in liquid nitrogen. These cases did not receive neoadjuvant therapy before operation. The samples were independently confirmed by two histopathologists, and were staged in line with the 8^th^ edition of AJCC [42].

### Cell lines

SMMC-7721, Huh7, HepG2, and L02 cell lines were acquired from the Chinese Academy of Sciences (Shanghai, China). MHCC-97H and MHCC-97L cells were originally acquired from the Fudan University (Shanghai, China). The details of cell culture medium as descried in our previous study [43]. All cells were deposited at 37° C in a humidified incubator comprising 5% CO_2_.

### Cell transfection and reagent

MiR-557 mimic, miR-557 inhibitor, miR-mimic negative control (NC), miR-inhibitor-NC, and the siRNA targeting RAB10 and NC (siRAB10, siNC) were acquired from RiboBio (Guangzhou, China). For RAB10 up-expression, cDNA of RAB10 was amplified by PCR and then inserted into the pcDNA3.1(+) plasmids (GenePharma, Shanghai, China). All of them were infected into cells with Lipofectamine 2000 (Invitrogen, USA). The infection rate was validated by RT-qPCR. The sequences of cDNA clone and siRNA are displayed in Supplementary Table 1.

### RNA extraction and RT-qPCR

TRIzol solution (Invitrogen, USA) was applied to abstract all RNA from cells and tissues in the light of the manufacturer’s protocols. The kits used for RT-qPCR as described in our previous study [43]. The relative levels of miR-557 and RAB10 were adjusted to U6 and β-actin, respectively. The RT and qPCR primers for miR-557 and U6 were acquired from RiboBio (Guangzhou, China). RAB10 primers refer to previous studies [44].

### Lentivirus construction and infection

We used lentiviruses to knock down and overexpress miR-557 in cell lines. The down-regulated and overexpression lentiviruses with a green fluorescent protein (GFP) for miR-557, and the matched control lentivirus were constructed by HANBIO (Shanghai, China). MHCC-97H or Huh7 cells were infected with above lentiviral vector for 24 h. The successfully infected HCC cells were filtered by 2 μg/ml puromycin. The infection efficiency was evaluated in each experiment by a fluorescence microscope and RT-qPCR. The stable-transfection cells were harvested for further experiments.

### Proliferation assay

The CCK-8 (Dojindo, Japan) solution was selected to assess the proliferative rate of HCC cells. Cells were placed in 96-hole plates at 5×10^3^ cells per hole. and 10 μL CCK-8 solution was mixed to each hole at 0, 24, 48, 72, and 96 h. Then incubated for a further 2 h. Absorbance was then gauged at 450 nm by the multimode reader (TECAN SPARK 10M, Switzerland).

### Colony formation assay

5×10^2^ HCC cells were placed in 6-hole plates and incubated for two weeks in the incubator. The plates were washed two times by PBS, then fastened by 4% paraformaldehyde and dyed with 1% crystal violet solution. The amount of cell colonies was counted by a microscope.

### Transwell assay

The 8 μm transwell chambers units (Corning, USA) were applied to assess the ability of cell invasion and migration. In the migration tests, 5×10^4^ cells were planted into the upper compartments in 200 μL DMEM reagent does not containing FBS, and 700 μL DMEM including 20% FBS was in the bottom compartments. For the invasion tests, 50 μL 1:8 Matrigel with DMEM (BD Biosciences, USA) was mixed into the upper compartments. The other steps were in light to the migration experiments. 24 h after cultured, the compartments were locked with 4% paraformaldehyde and dyed with 0.1% crystal violet solution. After that, the cells or Matrigel on the upper compartments were discarded. Cells were counted at five random area under a microscope (10×).

### Cell cycle analysis

HCC cells were placed into 6-hole plates. After infection for 48 h, cells were harvested by trypsin without EDTA and centrifuged (1×10^3^ r/min, 5 min). The cells were resuspended by precooling ethanol (70%) and incubated at 4° C overnight. Then, cells were flushed by precooling PBS two times and resuspended into a single cell suspension with 500 μL binding buffer. Each sample was incubated with 5 μL PI and 5 μL FITC Annexin (Beyotime, Shanghai, China) in the darkroom with room temperature for 15 min. The cell cycle stage was checked using BD FACSVerse™ flow cytometry (BD Biosciences, USA).

### The xenograft mice experiment

The animal tests were allowed by the Animal Care Commission of our center. All methods were reported in according with the recommendations of ARRIVE guidelines [45]. All methods were conducted conform to the relevant regulations and guidelines. The male BALB/c nude mice at 6-week-old were acquired from Hunan SJA Laboratory Animal C., Ltd. 5×10^6^ HCC cells infected with lentivirus in 100 μL DMEM reagent fixed with 50% Matrigel were subcutaneously injected into the nude mice on regions of right upper flank. The tumor width (W) and length (L) were gauged per three days. The formula ½×L×W^2^ was applied to gauge the tumor volume. After five weeks, the mice were sacrificed by cervical dislocation and tumors were excised for further study.

### Bioinformatics

The GEO online database was applied to explore the miR-557 expression in HCC. We downloaded GSE108724 including HCC and contiguous non-cancer tissues and GSE26323 including primary HCC tissues and pulmonary metastasis of HCC tissues. The differences in miR-557 expression between cancer and contiguous non-cancer tissues of the GEO datasets were calculated using the Limma package (version 3.22.4) of R software (version R 3.5.1) [46]. The original data needed logarithmic transformation followed by t-test. Adjusted *P*-values (adj. *P*) with default value was used to redress the potential false positive results. MiR-557 that met the cut-off criteria of adj. |log (fold change)|>2.0 combined with *P*<0.05 were considered to have a difference between tumor and non-tumor tissues.

The miR-557 target prediction algorithms TargetScan, miRTarBase, miRDB, and miRWalk were selected to calculate the downstream targets of miR-557. The Oncomine database, The UALCAN database, The Kaplan–Meier plotter online database was selected to further screening the downstream targets of miR-557.

The Oncomine database was applied to compare RAB10 expression between HCC and non-tumor samples. We meta analyzed four studies with the threshold as follow: fold change ≥ 2 combined with *P*-value ≤ 1E-4, top 10% gene.

The Human Protein Atlas database was applied to assess the differential RAB10 protein expression between HCC tumor and normal liver tissues.

The Kaplan–Meier plotter online database was applied to explore the prognostic impact of RAB10.

To investigate the potential pathogenic biological processes of RAB10, we conducted Gene Set Enrichment Analysis (GSEA) on TCGA-LIHC data using 4.1.0 GSEA software. The gene set of h.all.v7.4.symbols.gmt was chosen for further analysis. *P*<0.05 was considered to be significantly enriched.

### Luciferase reporter assay

The 3’-UTR including potential binding site (5’-TGCAAA-3’) of RAB10 for miR-557 (named WT-RAB10) and the corresponding mutation site (5’- GTAGCG-3’, named MT-RAB10) were duplicated and put into the pmirGLO Vector with Dual-Luciferase (Promega, USA). The pmirGLO-WT-RAB10 and pmirGLO-MT-RAB10 were co-infected with miR-557 mimics or miR-NC into MHCC-97H cells as described above. Then, Luciferase activities were checked and measured via the Dual-Luciferase reporter assay system in the light of the protocol from manufacturer.

The FOP-flash or TOP-flash with T-cell association with lymphoid enhancer factor (TCF/LEF) DNA combining loci were inserted into the reporter plasmids and the control plasmids pTK-RL. The plasmids were stem from Beyotime Biotechnology (Shanghai, China). The control and reporter plasmids were transfected into MHCC-97H cells in 24-hole plates through Lipofectamine 2000 reagent (Invitrogen, USA). After 48 h, the activity of luciferase was checked using Renila luciferase activity as the standard.

### Western blotting

RIPA buffer with protease inhibitors was selected to draw all proteins from HCC cells and patient tissues for further study. The NE-PER™ Cytoplasmic and Nuclear Extraction solutions (Thermo Fisher Scientific, USA) were selected to harvest proteins in nuclear. Protein concentrations were measured applying the protein quantitation kit (BCA, Pierce, USA). 30 μg of extracted protein was segregated by 8%–12% SDS-PAGE, and then shifted onto polyvinylidene fluoride (PVDF, Millipore, USA) membranes as soon as possible. All blots were cut prior to hybridisation with antibodies during western blotting. The PVDF membranes were intercepted with 5% skimmed milk at 37° C over 1 h, then hybridized in primary antibody solution overnight at 4° C. The dilution concentration of all primary antibodies was 1:1000 (E-cadherin #3195 CST; RAB10 ab237703 Abcam; Vimentin #5741 CST; MMP-9 ab76003 Abcam; MMP-2 ab92536 Abcam; N-cadherin #13116 CST; β-Catenin #8480 CST; Non-phospho (active) β-Catenin #19807 CST; GSK-3β #12456 CST; β-actin ab8226 Abcam; Phospho-GSK-3β (Ser9) #5558 CST; Histone H3 ab1791 Abcam). The PVDF membranes were then flushed three times by tris-buffered saline including Tween, and incubated with second antibody HRP-mouse (SA00001-1, 1:5000, Proteintech) or HRP-rabbit (SA00001-2, 1:5000, Proteintech) at 37° C for 1 h. Then, the intensity of bands was detected by ImageQuant LAS 500 chemiluminescence CCD (GE, USA) and gauged by ImageJ (NIH, USA).

### Statistical analysis

GraphPad Prism 7 and SPSS software (22.0) were selected to analyze the data. Mann–Whitney U-test or Student’s t-test was selected to compare continuous variables. Chi-square test, Spearman’s rank analysis, or Fisher’s exact test was selected to compare the independent samples and categorical variables. A two-tailed examine with *P*<0.05 was deemed statistically significant.

### Data availability

Access to the database can be obtained from the corresponding author on reasonable request.

## Supplementary Material

Supplementary Tables
