# Supplementary material for: miR-557 inhibits hepatocellular carcinoma progression through Wnt/β-catenin signaling pathway by targeting RAB10
Source: Aging (Albany NY). 2024 Feb 15;16(4):3716–33. doi: 10.18632/aging.205554 (PMC10929814; doi:10.18632/aging.205554)
Supplement: Supplementary Tables [file aging-16-205554-s001.pdf]

SUPPLEMENTARY TABLES

Supplementary Table 1. The sequences of siRNA and cDNA used in this study.

Name	Sequence
RAB10-shRNA	GCTGAAGATATCCTTCGAA
ORF Nucleotide Sequence of RAB10	ATGGCGAAGAAGACGTACGACCTGCTTTTCAAGCTGCTCCTGATCGGGGATTCCGGAG<br>TGGGGAAGACCTGCGTCCTTTTTCGTTTTTCGGATGATGCCTTCAATACTACCTTTATTT<br>CCACCATAGGAATAGACTTCAAGATCAAAACAGTTGAATTACAAGGAAAGAAGATCAA<br>GCTACAGATATGGGATACAGCAGGCCAGGAGCGATTTCACACCATCACAACCTCCTACT<br>ACAGAGGCGCAATGGGTATCATGCTAGTATATGACATCACCAATGGTAAAAGTTTTGAA<br>AACATCAGCAAATGGCTTAGAAACATAGATGAGCATGCCAATGAAGATGTGGAAAGAA<br>TGTTACTAGGAAACAAGTGTGATATGGACGACAAAAGAGTTGTACCTAAAGGAAAAGG<br>AGAACAGATTGCAAGGGAGCATGGTATTAGGTTTTTTGAGACTAGTGCAAAAGCAAATA<br>TAAACATCGAAAAGGCGTTCCTCACGTTAGCTGAAGATATCCTTCGAAAGACCCCTGTA<br>AAAGAGCCCAACAGTGAAAATGTAGATATCAGCAGTGGAGGAGGCGTGACAGGCTGG<br>AAGAGCAAATGCTGCTGA

Supplementary Table 2. The expression and survival analysis of miR-557 predicted target genes in hepatocellular carcinoma in oncimone, ualcan, and Kaplan Meier plotter databases.

GeneSymobl	Expression level in oncimone	Expression level in ualcan	Survival of ualcan database	Survival of Kaplan–Meier plotter database
USP6NL	P>0.05	P<0.05	P>0.05	P<0.05
FST	P>0.05	P<0.05	P>0.05	P>0.05
ARID1A	P<0.05	P<0.05	P<0.05	P<0.05
KCNIP3	P>0.05	P<0.05	P>0.05	P>0.05
RAPH1	P>0.05	P>0.05	P>0.05	P>0.05
WAC	P<0.05	P<0.05	P<0.05	P<0.05
LIMS1	P<0.05	P<0.05	P>0.05	P>0.05
CALM2	P<0.05	P<0.05	P<0.05	P<0.05
MAPK8	P>0.05	P<0.05	P>0.05	P>0.05
ACSL4	P<0.05	P<0.05	P>0.05	P>0.05
HBS1L	P>0.05	P<0.05	P>0.05	P>0.05
CDC42EP3	P>0.05	P<0.05	P>0.05	P<0.05
BCL7A	P<0.05	P<0.05	P<0.05	P>0.05
ADAM19	P>0.05	P<0.05	P>0.05	P<0.05
CRIPT	P<0.05	P<0.05	P<0.05	P>0.05
YIPF6	P<0.05	P<0.05	P>0.05	P>0.05
DUSP14	P>0.05	P<0.05	P<0.05	P>0.05
CLOCK	P<0.05	P<0.05	P>0.05	P>0.05
SESN3	P>0.05	P<0.05	P>0.05	P>0.05
TMEM245	Not found	gene symbols were not identified	gene symbols were not identified	The gene does not exist in database
UNKL	P>0.05	P<0.05	P<0.05	P<0.05
CBX4	P>0.05	P<0.05	P>0.05	P<0.05
JAZF1	P>0.05	P<0.05	P>0.05	P>0.05
RBPJ	P<0.05	P<0.05	P>0.05	P<0.05
REEP1	P>0.05	P<0.05	P>0.05	P<0.05
BACH2	P>0.05	P<0.05	P>0.05	P>0.05
ETS1	P>0.05	P>0.05	P>0.05	P<0.05
DNAJC15	P>0.05	P>0.05	P>0.05	P<0.05
IMPAD1	P<0.05	P<0.05	P>0.05	P>0.05
ZDHHC7	P>0.05	P<0.05	P<0.05	P<0.05
RAB10	P<0.05	P<0.05	P<0.05	P<0.05
ARHGEF3	P<0.05	P<0.05	P>0.05	P<0.05
CCNT2	P>0.05	P<0.05	P>0.05	P>0.05
ZBTB5	P<0.05	P<0.05	P>0.05	P>0.05
EOGT	Not found	gene symbols were not identified	gene symbols were not identified	The gene does not exist in database
GTF2H1	P<0.05	P<0.05	P<0.05	P<0.05
SATB1	P>0.05	P<0.05	P>0.05	P>0.05
SEMA6A	P>0.05	P<0.05	P<0.05	P<0.05
ST8SIA3	P>0.05	P<0.05	P>0.05	P>0.05
